# The Trickle-Down Effect of Territorial Behavior: A Moderated Mediation Model

**DOI:** 10.3389/fpsyg.2021.721806

**Published:** 2021-12-02

**Authors:** Yi Li, Haolin Weng, Ting Zhu, Na Li

**Affiliations:** School of Management, Shanghai University, Shanghai, China

**Keywords:** leader’s territorial behavior, employee’s territorial behavior, perceived insider status, team competitve climate, the trick-down effect

## Abstract

The present research seeks to explore how and when leader territorial behavior trickles down to the follower. Relying on social information processing theory, we hypothesize that territorial behavior has a trickle-down effect from leader to follower, and perceived insider status mediates the relationship between leader territorial behavior and follower territorial behavior. Competition climate is supposed to strengthen the effect of leader territorial behavior on perceived insider status. Two hundred and fifty-two dyads data of supervisor–subordinate in Chinese enterprises provided support for our hypotheses. The results suggest that leader territorial behavior is positively related to follower territorial behavior and that follower perceived insider status significantly mediates the relationship. Moreover, competition climate strengthens the negative relationship between leader territorial behavior and perceived insider status as well as the indirect effect of leader territorial behavior on follower territorial behavior *via* perceived insider status. Theoretical and practical implications are further discussed.

## Introduction

Territorial behavior has been widely discussed in the field of organization and management in recent years (Monaghan and Ayoko, 2019; Singh, 2019; Xu and Li, 2021). Territorial behavior refers to an individual’s behavioral expression of his or her feelings of ownership to a physical or social object (Brown et al., 2005). Historically, researchers focused on exploring the antecedents of territorial behavior such as individuals’ psychological ownership (Brown, 2009; Baer and Brown, 2012; Brown et al., 2014; Brown and Zhu, 2016; Wang et al., 2019), territorial infringement (Brown and Robinson, 2010), and organizational territorial climate (Li et al., 2020a), while surprisingly scarce researches have been done about leadership factors as antecedent (for exceptions, see Brown and Menkhoff, 2007; Brown and Zhu, 2016; Boekhorst et al., 2019). Besides, relatively few studies examined the leader territorial behavior (Gardner et al., 2016; Zhu et al., 2021). Such an omission is surprising given that the supervisor serves as the agent representing the organization (Coyle-Shapiro and Shore, 2007) and supervisor territorial behavior may have great influence on the whole organization (Brown and Menkhoff, 2007). Following this logic, it is important to consider whether there will be a trickle-down effect of leader territorial behavior on follower territorial behavior.

Social information processing model indicates that individuals make decisions and exhibit subsequent behaviors according to the relevant information that they obtain from their surroundings (Salancik and Pfeffe, 1978). Supervisors, as significant clues of organizational environment, are critical to employee’s perception and behaviors (Bavik et al., 2018). Perceived insider status is indicative of a sense of belonging within the organization (Masterson and Stamper, 2003), describing the extent to which an employee perceived himself/herself as an insider in a particular organization (Stamper and Masterson, 2002). In fact, for employees, leaders are the main transmitters of information, and they are entitled to control employees’ resources, salary, and career development (Dépret and Fiske, 1993; Hu and Shi, 2015). Individuals thus form perceptions about their status as an organizational member by the information or clues from their leaders’ behaviors (Stamper and Masterson, 2002; Loi et al., 2012). Thus, we propose that supervisor’s territorial behaviors can reduce perceived insider status of employees and further increase their territorial behaviors.

The influence of supervisor’s territorial behavior on employees’ perceived insider status may not always exist and may be affected by organizational contextual factors. Therefore, we infer that there is a boundary condition on the relationship between leader’s territorial behavior and follower territorial behavior. We contend that a key aspect of the social work environment that reflects personal relationships with relevant others is the degree of competition in the environment. Competition has been considered as a situation where individuals vie for limited resources or rewards (Wang et al., 2018). Competition climate may increase pressure and reduce team cooperation (Connelly et al., 2017; David et al., 2021). Thus, we argue that competitive climate prompts employees to pay more attention to the relationship between the leader and the resources provided by the leader. Following this rationale, we posit that competitive climate may moderate the relationship between supervisor’s territorial behavior and perceived insider status. By examining the moderation effect of competitive climate and the mediation effect of perceived insider status, we can further clarify the conditions under which territorial behavior can trickle down from leaders to employees.

**Figure 1:**
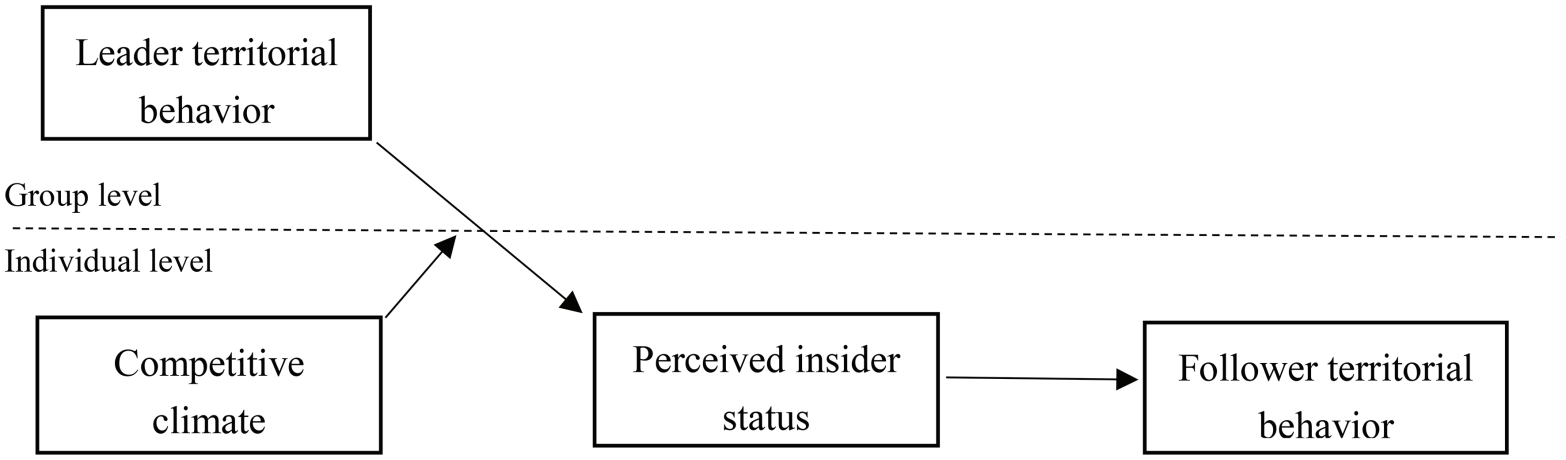
Theoretical model.

This study contributes to the literature in several ways. First, we attempt to increase our understanding of territorial behavior literature by demonstrating the trickle-down effect of territorial behavior. Some studies have explored the territorial behavior and its impact at the individual level (Boekhorst et al., 2019; Zhu et al., 2021). However, the effect of leader territorial behavior is still unexplored. We attempt to address this gap in the literature by examining the trickle-down effect of leader territorial behavior. Second, social information processing model is one of the main theories in the trickle-down model and has been widely used in trickle-down phenomena (Loi et al., 2012; Vlachos et al., 2014). This study attempts to explain the mediating role of perceived insider status, which will help to explain the mechanism by which supervisor’s territorial behavior affects employee’s territorial behavior, offering fresh insights into territorial behavior research. Our theoretical model is summarized in Figure 1.

## Theories and Hypotheses

### The Trick-Down Effect of Territorial Behavior

Territorial behavior refers to behaviors that individuals used to mark and defend the social resources who feel ownership, including tangible resources such as physical space and possessions, as well as intangible resources, such as information and relationships (Gardner et al., 2016). In organization, leaders generally have higher positions than employees have in the organizational hierarchy, possess more valued resources (e.g., spaces, roles, relationships, responsibilities, knowledge, experiences, even the employees) and control the resources allocation within the team (Brown et al., 2005; Chen et al., 2019). Therefore, leaders may engage in territorial behavior because of the social defined nature of territoriality. Examples of leader territorial behavior might include a nameplate on the door or the titles like ‘Manager’ and ‘Lead’ to express their identity and a proprietary space in a shared office or the efforts to stop employees from accessing to important information (Brown et al., 2005).

As Friedkin (2001) suggested, the norms formed through a process of interpersonal influence with leaders who have influential positions. This is because leaders can transmit the accepted norms and values to the team members through the way they behave (Thom-Santelli, 2009). Therefore, leader territorial behavior may lead to the formation of team territorial norms and further influence whether an individual will engage in territorial behavior and the degree involved (Brown and Zhu, 2016). In such territorial norms, team members may protect their territories, maintaining territorial boundaries and be reluctant to venture into certain areas, take on certain roles, or establish certain relationships out of respect for another’s ownership of those territories (Brown et al., 2005). Consequently, individuals isolate themselves from others, neglect their relationship to the organization, and focus on their territories. We then predict that territorial behavior could trickle down from leaders to followers.

*H1*: Leader territorial behavior is positively related to follower territorial behavior.

### Leader Territorial Behavior and Perceived Insider Status

The perceived insider status describes the extent to which an employee perceived himself/herself as an insider in a particular organization (Stamper and Masterson, 2002), and it connotes an employees’ sense of having earned a personal space and acceptance as an organizational member (Masterson and Stamper, 2003). Social information processing theory suggests that social information people get from work environment can affect people’s perceptions, attitudes, and behavior (Zalesny and Ford, 1990). Employees tend to collect relevant information from what their leader do and say to shape their perceptions and behaviors (Hu and Shi, 2015). Therefore, as the representative of the organization, the leader usually provides employees with relevant social cues about their status as an organizational member (Stamper and Masterson, 2002; Loi et al., 2012). This reasoning is consistent with the relational model of authority proposed by Tyler and Lind (1992), who suggested that perceptions of one’s relation to an authority are essential indicators of one’s relation to the entire group, the employees’ feeling about how ‘included’ they are in the organization may, therefore, depend on how they are treated by the supervisor.

As a leader behavior, the leader’s behavioral expression of his or her territory is the important social cues that employees may use to interpret their organization membership. Territorial behavior may affect others’ perceptions of the individual who engage in territorial behavior. For example, territorial behavior may be viewed as an attempt to control resources (Brown and Zhu, 2016); individuals involved in territorial behavior may be considered as an uncooperative person. Therefore, leader territorial behavior may adversely, and perhaps unintentionally, send negative information to others by protecting valued resources and sharing less information, thereby employees may feel that they received less support and lower trust from the leader and organization. Given that the perceived organizational/leader support and trust are important factors influencing perceived insider status (Lapalme et al., 2009), leader territorial behavior may harm employees’ perceived insider status.

Moreover, as a social-behavioral construct, territoriality, in particular, affects the interactions between members in the organization (Webster et al., 2008). Hence, leader territorial behavior forms negative interaction between leaders and employees. Brown and Menkhoff (2007) have suggested that the result of leader territorial behavior makes employees increasingly frustrated with their treatment and lack of acknowledgment, further affecting the leader–follower relationship and organization–follower relationship and reducing employees’ sense of belonging and loyalty to the organization. Considering perceived insider status is a product of employees’ sense-making processes that derive from inputs such as high-quality work relationships. We therefore predicted:

*H2*: Leader territorial behavior is negatively related to follower perceived insider status.

### Perceived Insider Status and Employee Territorial Behavior

As a reflection of the quality of employee–organization relations, employees’ perceived insider status is a dimension to measure employees’ sense of belonging (Masterson and Stamper, 2003), which refers to a type of personal perception of being a member of an organization. Employees who perceived themselves as insiders in the organization are more likely to form the cognition of citizens of the firm and accept the role, responsibilities, and requirements consistent with this identity (Hui et al., 2015). Therefore, as important members of an organization, employees will share their resources and invest more resources in defending the organization (Lapalme et al., 2009) and increase their participation and effort to help the organization (Han et al., 2010). In contrast, if the employees consider themselves as outsiders of the organization members, individuals will be more interested in preserving their own interests and less concerned about the welfare of others or the entire team (Brown et al., 2014). We thus propose:

*H3*: Perceived insider status is negatively related to follower territorial behavior.

### The Mediation of Perceived Insider Status

The present research suggests that leader territorial behavior leads to follower territorial behavior because it reduces a sense of insider membership in the organization. According to the research of Lapalme et al. (2009), when the organization limits its investment in the employees, they may develop a perception that they are outsiders, these employees then limit their investment in the organization. Therefore, leader territorial behavior can reduce knowledge sharing and decrease resources allocation, which sends signals that indicate the individual does not matter to the company. Such a leader’s behavior reduces employee perceived insider status. Subsequently, the employees will seek less interaction with organization members, reinforce self-protection, and reduce resource sharing. We then predict:

*H4*: Perceived insider status mediates the relationship between leader territorial behavior and employee territorial behavior.

### The Moderation of Competitive Climate

Competitive climate represents the extent to which employees perceive organization rewards to be contingent on comparisons of their performance against that of their peers (Brown et al., 1998). Like any environmental context, a competitive environment can have a significant influence on relationships between variables (Johns, 2006). This is because it is part of employees’ sense-making, helping them to both construct and interpret events that happen in that environment (Salancik and Pfeffe, 1978). Therefore, we argued that as a contextual factor in organizations, competition climate serves a critical role that moderates the impact of leader territorial behavior on employees perceived insider status by influencing how individuals understand their relationship with others.

First, by definition, competitive psychological climate consists of the following aspects: perceptions of differential reward distribution, the performance compared to other individuals, perceived competition with others, and frequent status comparisons (Fletcher et al., 2008). The comparisons with other employees cause further stress to the individual (Arnold et al., 2009), reduce collaboration with team members, and even lead to ostracizing (Ng, 2017). As a result, highly competitive climate destroys the trust foundation among team members, reduces their quality of the relationship, and makes employees pay more attention to the relationship with leaders. As a negative interpersonal interaction, leader territorial behavior will have a stronger impact on employees’ perception. In addition, perceptions of competitive climate reflect employees’ sense of the extent to which their job rewards, promotion, and retention depend on performance compared to others (Brown et al., 1998). Given that the important role of leaders in employee performance evaluation and career development, employees are sensitive to their leaders’ evaluation. More importantly, from the perspective of limited resources, the competitive climate describes a situation where individuals or organizations vie for limited resources or rewards (Wang et al., 2018). To access resources and achieve high performance, the employees focus on the leaders’ attitudes and behaviors. Therefore, when leaders engage in territorial behaviors, employees have a stronger reaction to leaders’ negative interpersonal treatment in a higher competitive climate. We then predict:

*H5*: Competitive climate moderates the negative relationship between leader territorial behavior and perceived insider status, such that the negative relationship is stronger when competitive climate is higher.

Taken as a whole, the hypotheses presented above imply a moderated mediation model. Competitive climate may moderate the indirect effect of leader territorial behavior on employee territorial behavior through employee perceived insider status. Perceived insider status explains the relationships between leader territorial behavior and employee territorial behavior (H4), but because the relationship between leader territorial behavior and perceived insider status is predicted to be stronger when the competitive climate is higher (H5), we predict that the mediated relationships captured by Hypothesis 4 are stronger when the competitive climate is higher. We then predict:

*H6*: Competitive climate moderates the indirect relationships between leader territorial behavior and employee territorial behavior such that the indirect effects are stronger when competitive climate is higher.

## Materials and Methods

### Sample and Procedure

We tested our hypotheses with data collected from three enterprises in Shanghai. To reduce common method variance and illusionary correlations, we collected data in two waves from May to June 2020. In the first stage, the leaders were asked to rate their territorial behavior and provided information in relation to their demographics, and the employees rated team competitive climate and provided information in relation to their demographic. Prior permission from HR departments in these enterprises was sought, and they also assisted us in survey distribution. To perform dyadic matching between employees and their corresponding leaders, all respondents were asked to indicate their leader or subordinates in the enterprises where they work. We explained the purpose of the research, emphasizing that the research is only for scientific study purposes, besides, the questionnaire number and personnel code were issued in a one-to-one correspondence way to ensure the authenticity, confidentiality, and accuracy of the questionnaire survey. One month later, the employees who responded in phase one were asked to rate their perceived insider status and territorial behavior online.

A total of 380 dyads questionnaires were distributed. After eliminating the obviously invalid questionnaires, the final sample of 252 employees with 65 managers was retained for a total response rate of 66.32%. Of those participants, the average income was 7.31 thousand yuan (SD=3.11); 64 percent were women (SD=0.48), and they averaged 24.47years of staying at the company (SD=22.28).

### Measures

The instruments were administered in Chinese in our survey but were originally developed in English. To confirm the accuracy of the translation and correct any discrepancies, we employed back-translation procedures (Brislin, 1986). Unless otherwise indicated, we used a five-point Likert-type scale, ranging from 1 (strongly disagree) to 5 (strongly agree).

#### Territorial Behavior

Territorial behavior (leader and follower) was measured using the six-item scale developed by Brown et al. (2014). A sample item is ‘I hide the ‘work details or tricks’ so others do not know about it until I want.’ Cronbach’s alpha of the leader scale was 0.85, and the employee scale was 0.82.

#### Perceived Insider Status

Perceived insider status was measured using the six-item scale developed by Stamper and Masterson (2002). A sample item is ‘My work organization makes me believe that I am included in it.’ Cronbach’s alpha of the scale was 0.82.

#### Competitive Climate

For the measurement of competitive climate, we used the four-item scale of perceived team competitive climate by Brown et al. (1998). A sample item is ‘My coworkers frequently compare their results with mine.’ Cronbach’s alpha of the scale was 0.71.

#### Control Variables

Previous studies have shown that certain socio-demographic variables like gender can affect territorial behavior (Mercer and Benjamin, 1980). Therefore, we controlled for the income and gender. We further controlled for the job tenure of subordinates because it takes some time to establish a supervisor–subordinate relationship.

## Results

### Descriptive Statistics and Correlations

Table 1 shows the means, standard deviations, and correlation coefficients of the variables. The correlations are as expected. Leader territorial behavior was positively correlated with employee territorial behavior (*γ*=0.23, *p*<0.001). Leader territorial behavior was negatively correlated with perceived insider status (*γ*=−0.25, *p*<0.001). Perceived insider status was negatively correlated with employee territorial behavior (*γ*=−0.33, *p*<0.001).

**Table 1:** Means, standard deviations, and correlations among variables.

Variable	Mean	SD	1	2	3	4	5	6	7
1. Monthly income before tax	7.31	3.11							
2. Gender	1.64	0.48	−0.17^**^						
3. Tenure	24.47	22.28	0.23^**^	−0.04					
4. Leader territorial behavior	2.51	0.86	0.13^*^	−0.12	−0.03	(0.85)			
5. Perceived insider status	3.88	0.71	0.06	0.15^*^	0.02	−0.25^***^	(0.82)		
6. Team competitive climate	3.15	0.77	0.13^*^	−0.16^*^	0.07	0.06	−0.09	(0.71)	
7. Employee territorial behavior	2.50	0.78	0.21^**^	−0.21^**^	0.12	0.23^***^	−0.33^***^	0.34^**^	(0.82)

^*^p<0.05; ^**^p<0.01; ^***^p<0.001. The alpha reliability coefficient of each variable is in brackets. Income: measured in thousands of Yuan; Gender: 1=male; 2=female. Means, standard deviations, and correlations among variables.

### Reliability and Validity

This study performed Harman’s one-factor test to verify the risk of common method effect (Podsakoff et al., 2003), which indicated that Harman’s single-factor test indicates the fixed single factor explains 25.28 percent of the covariance of the variables. The reliability of the multi-item scale for each dimension was assessed by using Cronbach’s alpha coefficient. The results in Table 2 showed that Cronbach’s alpha values of all of the constructs ranged from 0.71 to 0.85, exceeding the recommended minimum standard of 0.70 (Fornell and Larcker, 1981). Besides, the results in Table 3 showed that the composite reliability (CR) is higher than 0.7. Therefore, the reliability of the measurement in this study was acceptable.

**Table 2:** Confirmatory factory analysis results.

Models	*χ* ^2^	df	*χ*^2^/df	TLI	CFI	RMSEA	SRMR	∆*χ*^2^
Four-factor model	151.52	48	3.16	0.90	0.93	0.09	0.06	
Three-factor model	394.88	51	7.74	0.68	0.75	0.16	0.15	243.34
Two-factor model	583.03	53	11.00	0.53	0.62	0.20	0.18	431.51
One-factor model	770.65	54	14.27	0.37	0.49	0.23	0.17	619.13

*Model with three factors: (1) leader territorial behavior+perceived insider status, (2) team competitive climate, and (3) employee territorial behavior. Model with two factors: (1) leader territorial behavior+perceived insider status+team competitive climate and (2) employee territorial behavior. Model with one factor: all items combined with one factor*.

**Table 3:** Convergent validity.

Variable	Item	Factor loading	AVE	CR
Leader territorial behavior	1. I hide the ‘work details or tricks’ so others do not know about it until I want.	0.54	0.51	0.86
	2. I let others know the ‘work details or tricks’ has been claimed.	0.59		
	3. I tell/ show others that the ‘work details or tricks’ belongs to me.	0.68		
	4. I clarify the boundaries around the ‘work details or tricks’ (to establish what is and is not yours).	0.75		
	5. I make the ‘work details or tricks’ hard to use/ access.	0.84		
	6. I make the ‘work details or tricks’ unattractive so others do not want to claim it.	0.85		
Employee territorial behavior	1. I hide the ‘work details or tricks’ so others do not know about it until I want.	0.57	0.50	0.85
	2. I let others know the ‘work details or tricks’ has been claimed.	0.59		
	3. I tell/ show others that the ‘work details or tricks’ belongs to me.	0.59		
	4. I clarify the boundaries around the ‘work details or tricks’ (to establish what is and is not yours).	0.79		
	5. I make the ‘work details or tricks’ hard to use/ access.	0.84		
	6. I make the ‘work details or tricks’ unattractive so others do not want to claim it.	0.79		
Perceived insider status	1. I do not feel included in this organization.	0.66	0.51	0.86
	2. I feel like I am an ‘outsider’ at this organization.	0.75		
	3. My work organization makes me believe that I am included in it.	0.74		
	4. I feel I am an ‘insider’ in my work organization.	0.80		
	5. My work organization makes me frequently feel ‘left-out’.	0.49		
	6. I feel very much a part of my work organization.	0.81		
Team competitive climate	1. My coworkers frequently compare their results with mine.	0.75	0.50	0.79
	2. The amount of recognition you get in this company depends on how your work performance rank compared to other coworkers.	0.75		
	3. Everybody is concerned with finishing at the top of the performance rankings.	0.59		
	4. My manager frequently compares my results with those of other coworkers.	0.73		

In addition, we computed the average variance extracted (AVE) for all variables. Discriminant validity was established by ensuring AVEs of any two variables which were higher than the square of their correlations (Fornell and Larcker, 1981; Wang et al., 2021). In other word, the square root of AVEs of the variable is greater than the correlation coefficient between the variable and other variables, thus confirming the discriminant validity. The results in Table 4 showed that this rule was not violated as the inter-construct correlation coefficients ranged from 0.06 to 0.34, whereas the square root of the AVEs is 0.71, indicating acceptable discriminant validity.

**Table 4:** Correlation and the square roots of AVEs.

	Leader territorial behavior	Perceived insider status	Team competitive climate	Employee territorial behavior
Leader territorial behavior	**0.71**			
Perceived insider status	−0.25	**0.71**		
Team competitive climate	0.06	−0.09	**0.71**	
Employee territorial behavior	0.23	−0.33	0.34	**0.71**

*Square roots of AVE are in bold prints in the diagonal; inter-construct correlation coefficients are in the left lower half*.

The results in Table 3 showed that all the items loaded significantly onto their correspondent constructs with the factor loading range from 0.49 to 0.85, and average variance extracted (AVE) is higher than 0.5, indicating acceptable convergent validity. Although most items loaded nicely on their respective factors with standardized loadings coefficients being from 0.49 to 0.85, some items still loaded low (<0.708). We used full items in data analysis. This is because they are original measurement items for the internalization dimension of four scales, and the development of these items has undergone a rigorous psychometric process (Brown et al., 1998; Stamper and Masterson, 2002; Brown et al., 2014). Meanwhile, the reliability of the full item measure (>0.70) is adequate for research (cf. Nunnally, 1978; Hair et al., 2010) and consistent with those reported in other studies including that of Brown and Zhu themselves (2016; for others see Xiong Chen and Aryee, 2007; Chen et al., 2015; Wang et al., 2019).

We performed a confirmatory factor analysis (CFA) using Mplus7.4 to compare possible measurement models. The results in Table 2 showed that the proposed four-factor model demonstrated a better fit (*χ*^2^=151.52, df=48, RMSEA=0.09, CFI=0.93, TLI=0.90, SRMR=0.06) to the data than other alternative models, indicating support for the distinctiveness of the constructs in the study. These results proved that the four-factor model was the most appropriate one that provided support for the convergent validity of our variables.

### Null Model

We calculated the ICC(1) for employee territorial behavior to ascertain whether the use of multilevel modeling is necessary to analyze our data (Stawski, 2013). The ICC(1) was 0.12, meaning that 12% of the overall variance in employee territorial behavior was due to differences between groups, thus warranting a multilevel approach to data analysis.

### Hypothesis Testing

Hypothesis 1 predicted a positive relationship between leader territorial behavior and follower territorial behavior. In Model 3 of Table 5, the results suggested that leader territorial behavior was positively related to follower territorial behavior (*γ*=0.22, *p*<0.01), supporting Hypothesis 1.

**Table 5:** Results of multiple regression analysis.

Variables	Perceived insider status				Employee territorial behavior					
	Model 1		Model 2		Model 3		Model 4		Model 5	
	γ	S.E.	γ	S.E.	γ	S.E.	γ	S.E.	γ	S.E.
Intercept	3.66^***^	0.54	4.16^***^	0.22	2.35^***^	0.61	3.80^***^	0.33	4.49^***^	0.79
**Control variables**										
Monthly income before tax	0.06	0.04	0.02	0.01	−0.01	0.05	0.05^**^	0.02	0.02	0.04
Gender	0.24	0.20	0.17^*^	0.08	−0.27	0.17	−0.20^*^	0.10	−0.18	0.15
Tenure	−0.01	0.00	0.00	0.00	0.01	0.00	0.00	0.00	0.00	0.00
**Level 1 variables**										
Perceived insider status							−0.36^***^	0.07	−0.56^**^	0.17
Level 2 variable										
Leader territorial behavior	−0.22^**^	0.06	−0.19	0.06	0.22^**^	0.07			0.08	0.06
Competitive climate			0.18	0.03						
**Cross-level interaction variable**										
Leader territorial behavior ^*^competitive climate			−0.22^***^	0.09						
*Pesduo-R^2^*		0.18				0.07			0.22	

^*^p<0.05; ^**^p<0.01; ^***^p<0.001.

Hypothesis 2 posited that leader territorial behavior is negatively related to perceived insider status, and Hypothesis 3 proposed a negative relationship between perceived insider status and follower territorial behavior. As shown in Table 5, the results of Model 1 revealed that leader territorial behavior had a significant negative effect on perceived insider status (*γ*=−0.22, *p*<0.01). Thus, Hypothesis 2 was supported. The results of Model 4 revealed that perceived insider status had a significant negative effect on follower territorial behavior (*γ*=−0.36, *p*<0.001). Thus, Hypothesis 3 was supported. Further, in Model 5, after entering perceived insider status, the positive relationship between leader territorial behavior and follower territorial behavior was not significant (*γ*=0.08, *p*>0.05). According to Baron and Kenny procedures (Baron and Kenny, 1986), we found support for the mediation of perceived insider status. In addition, we used the Monte Carlo simulation approach (Preacher and Selig, 2012) to assess the indirect effect. The results showed that the indirect effect was significant [95% CI=(0.01, 0.23), excluding 0]. Thus, Hypothesis 4 was supported.

**Figure 2:**
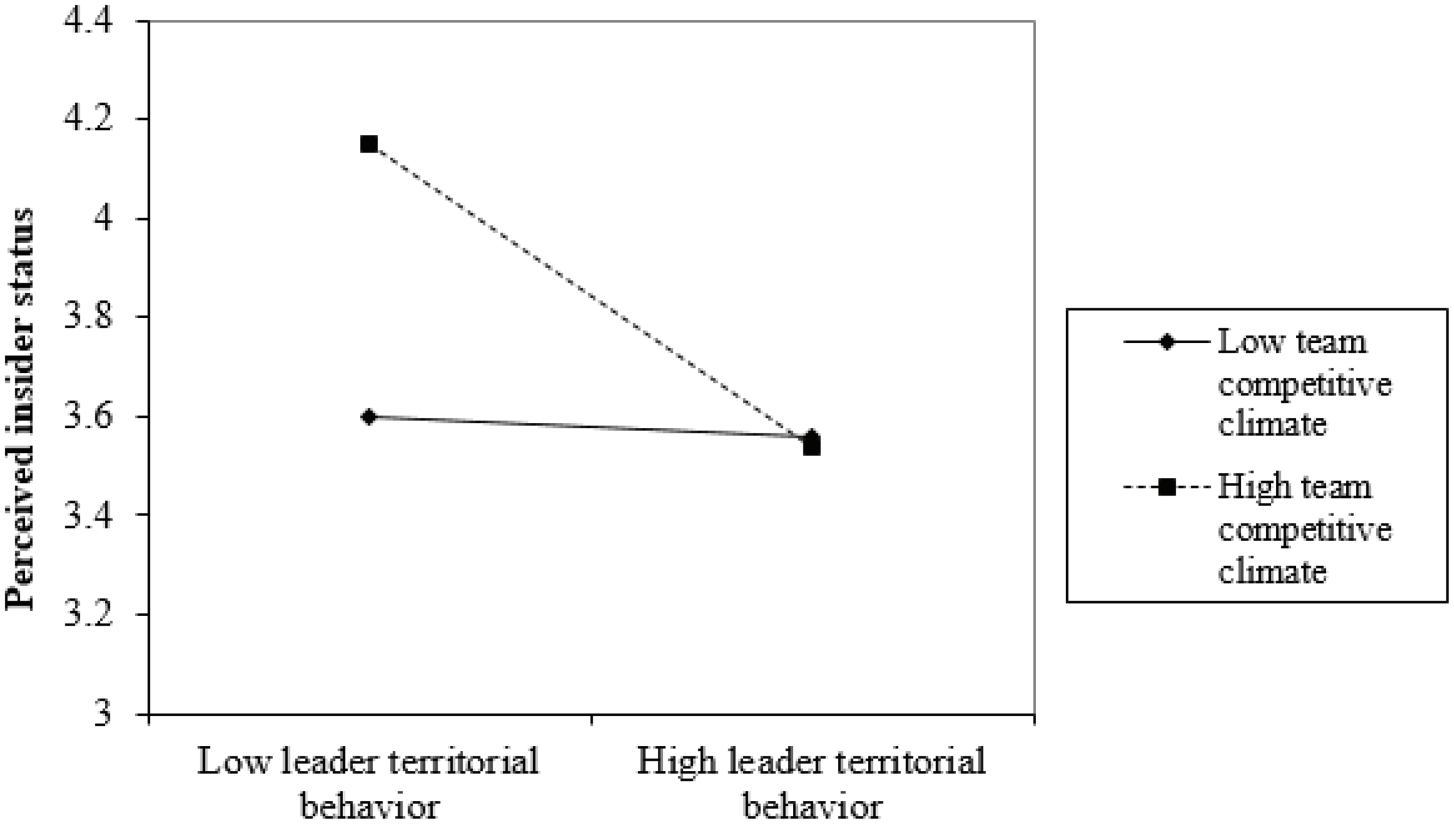
Effect of the interaction between leader’s territorial behavior and team competitive climate on employee’s perceived insider status.

Hypothesis 5 predicted that competitive climate would moderate the relationship between leader territorial behavior and perceived insider status. As shown in Model 2, the interactive effect was significant (*γ*=−0.22, *p*<0.001). Figure 2 further showed that this relationship was more negative when competitive climate was high (one SD above the mean) rather than low (one SD below the mean). Thus, Hypothesis 5 was supported.

Hypothesis 6 predicted that competitive climate would moderate the indirect effect of leader territorial behavior on follower territorial behavior through perceived insider status. According to results presented in Table 6, competitive climate significantly moderated this indirect effect [difference=0.15, *p*<0.05, 95%CI=(0.01, 0.29), excluding 0]. Specifically, when competitive climate was high (one SD above the mean), moderated mediation effect was 0.10 [*p*<0.05, 95%CI=(0.01, 0.19), excluding 0]; when competitive climate was low (one SD below the mean), the moderated mediation effect was not significant. Thus, Hypothesis 6 was supported.

**Table 6:** Results of the moderated mediation effect.

	γ	SE	95% confidence interval	
			Lower limit	Upper limit
High competitive climate (+1 SD)	0.10	0.05	0.01	0.19
Low competitive climate (−1 SD)	−0.05	0.04	−0.12	0.03
Difference between two groups	0.15	0.07	0.01	0.29

## Discussion

The main objective of the present research is to explore how and when leader territorial behavior trickles down to followers. Relying on social information processing theory, we explored the mediating role of perceived insider status in linking leader territorial behavior with employee territorial behavior and the moderating role of competitive climate in influencing the relationship between leader territorial behavior and perceived insider status. As hypothesized, we found that leader territorial behavior was positively related to employee territorial behavior and that perceived insider status mediated the relationship. Moreover, competitive climate strengthened the negative relationship between leader territorial behavior and perceived insider status and the indirect effect of leader territorial behavior on employee territorial behavior *via* perceived insider status. We now discuss the theoretical and practical implications of the results.

### Theoretical Implications

Our research provides empirical evidence for the trickle-down effect of a leader’s territorial behavior on an employee’s territorial behavior within the social information processing theory framework. Specifically, we are the first to theorize and propose a model in which leader’s territorial behavior trickles down to employee in the organization, which respond to Thom-Santelli’s (2009) call for exploring the predictors of territorial behavior from the perspective of leader. Our study’s results not only support the view that ‘Leaders are an important factor influencing follower behaviors and perceptions’ (Ambrose et al., 2013; Bavik et al., 2018; Chen et al., 2019), they also supplement the literature on trickle-down effects of leader behaviors (e.g., Frazier and Tupper, 2016; Lu et al., 2018; Chen et al., 2019; Byun et al., 2020; Zhang et al., 2020). Previous studies primarily focused on the territorial behavior and its impact at the individual level (Boekhorst et al., 2019; Zhu et al., 2021), while surprising few researches have been done about leader territorial behavior (for exceptions, see Gardner et al., 2016). There is territoriality at different levels of the organization (Brown et al., 2005). Given the territorial nature of human beings and the particular status of leaders in the organization, it is not surprising that leaders engage in territorial behavior. Gardner et al.’s (2016) study showed that managers engaged in territorial behaviors to maintain ownership claims over their employees. In addition, some studies suggested that leaders will protect the information and relationship, which affect the organization–employee relationship (Brown and Menkhoff, 2007). Therefore, this research contributes to the understanding of the leader territorial behavior and its consequences by describing how leader territorial behavior promotes employee territorial behavior on the organization.

Another contribution is our exploration of the trickle-down effect mechanism through which the effects of leader territorial behavior trickle down to employee territorial behavior. In doing so, we respond to scholars’ calls for more investigations to open the black box of the influences of leader territorial behavior (Gardner et al., 2016) by providing empirical evidence of employee perceived insider status as a mediator. Moreover, we provide a new perspective for the study of territorial behavior–social information processing theory. Specifically, our research differs from existing territorial behavior literature, which is largely based on the extended self-theory (Wang et al., 2019) and social exchange theory (Huo et al., 2017; Singh, 2019; Li et al., 2020b). In doing so, we extend the application of social information processing theory in the territorial behavior literature.

### Practical Implications

Our findings have several important practical implications. First, the present study found that leader territorial behavior has trickled down effects on followers. Thus, organizations should take measures to reduce leader territorial behavior in the workplace. For example, the organization can effectively inhibit the leader territorial behavior by building an open office environment, encouraging leadership delegation, and holding experience sharing sessions. Second, the findings showed that perceived insider status mediates the trickle-down process. Thus, organizations can reduce employee territorial behavior by taking measures to increase employee perceived insider status. In the business management practice, organizations should offer resources support to their employees, who need to feel that they are part of group members or have special status. Moreover, organizations could also enhance employees’ perceived insider status through other ways such as delegation or organizational inducements (Hui et al., 2015). Third, the present study found that competitive climate strengthens the link between leader territorial behavior and perceived insider status. Thus, organizations should create a healthy competition atmosphere and avoid excessive competition to weaken the negative effect of leader territorial behavior on employee territorial behavior.

### Limitations and Future Research

Our research has some limitations that should be acknowledged.

First, our method is restricted in some respects. Our data fitting results are acceptable, but still not good enough, such as RMSEA=0.09 and AVE=0.50 are slightly higher than the acceptable range when other indicators are acceptable. We think there may be two reasons. On the one hand, according to Bandalos’s recommendation (Bandalos, 2002), the parameter-to-item ratio should be above almost a certain proportion (10:1), our sample size is slightly higher than the acceptable standard, the direct use of the original title may lead to some estimation bias, and on the other hand, the reason why the Cronbach’s alpha of the competitive climate scale is 0.71 is that the participants may not be willing to truly evaluate the competitive atmosphere. Although this reliability is acceptable, it may still affect the fit of the whole model. Therefore, future research should use a more perfect questionnaire process to ensure that the participants can be express their real ideas and verify the conclusion with a larger sample. Besides, our three-waved time-lagged data still cannot verify causality certainly for all variables in our model. Future research should consequently replicate our conclusions with a more rigorous longitudinal research method or experimental method.

Second, samples from Chinese enterprises limit the generalizability of the findings to different contexts. Cultural values can influence how individuals perceive and react to leader behavior (Peng and Kim, 2020; Zhang et al., 2021). Therefore, further research could examine the relationship between leader territorial behavior and follower territorial behavior in other cultural contexts.

Third, we explored only one boundary condition—competitive climate moderates the relationship between leader territorial behavior and employee territorial behavior. There may be other moderator variables to mitigate the negative impact of leader territorial behavior, and future research can explore other organizational factors to reduce the negative impact of leader territorial behavior.

## Conclusion

From the perspective of social information processing, this paper expounds in detail that territorial behavior has a trickle-down effect from leader to follower and perceived insider status mediates the relationship between leader territorial behavior and follower territorial behavior. Our findings expand the perspective of territorial behavior research and hope to spark further research on territorial behavior.

## Data Availability Statement

The original contributions presented in the study are included in the article/Supplementary Material, further inquiries can be directed to the corresponding author.

## Author Contributions

All authors listed have made a substantial, direct and intellectual contribution to the work, and approved it for publication.

## Conflict of Interest

The authors declare that the research was conducted in the absence of any commercial or financial relationships that could be construed as a potential conflict of interest.

## Publisher’s Note

All claims expressed in this article are solely those of the authors and do not necessarily represent those of their affiliated organizations, or those of the publisher, the editors and the reviewers. Any product that may be evaluated in this article, or claim that may be made by its manufacturer, is not guaranteed or endorsed by the publisher.
