# Supplementary material for: The Trickle-Down Effect of Territorial Behavior: A Moderated Mediation Model
Source: Front Psychol. 2021 Dec 2;12:721806. doi: 10.3389/fpsyg.2021.721806 (PMC8675102; doi:10.3389/fpsyg.2021.721806)
Supplement: Supplementary file 1 [file Data_Sheet_1.ZIP › factor load.AmosOutput]

1.amw


#### C:\Users\Weng Haolin\Desktop\李娜paper\数据\调的amos\1.amw

##### Analysis Summary

##### Date and Time

Date: 2021年10月1日

Time: 4:03:30

##### Title

1: 2021年10月1日 4:03

##### Groups

##### Group number 1 (Group number 1)

##### Notes for Group (Group number 1)

The model is recursive.

Sample size = 252

##### Variable Summary (Group number 1)

##### Your model contains the following variables (Group number 1)

Observed, endogenous variables

y12

y11

y10

y9

y8

y7

y18

y17

y16

y15

y14

y13

y6

y5

y4

y3

y2

y1

y22

y21

y20

y19

Unobserved, exogenous variables

F1

e1

e2

e3

e4

e5

e6

F2

e7

e8

e9

e10

e11

e12

F3

e13

e14

e15

e16

e17

e18

F4

e19

e20

e21

e22

##### Variable counts (Group number 1)

|  |  |
| --- | --- |
| Number of variables in your model: | 48 |
| Number of observed variables: | 22 |
| Number of unobserved variables: | 26 |
| Number of exogenous variables: | 26 |
| Number of endogenous variables: | 22 |

##### Parameter Summary (Group number 1)

|  | Weights | Covariances | Variances | Means | Intercepts | Total |
| --- | --- | --- | --- | --- | --- | --- |
| Fixed | 26 | 0 | 0 | 0 | 0 | 26 |
| Labeled | 0 | 0 | 0 | 0 | 0 | 0 |
| Unlabeled | 18 | 6 | 26 | 0 | 0 | 50 |
| Total | 44 | 6 | 26 | 0 | 0 | 76 |

##### Models

##### Default model (Default model)

##### Notes for Model (Default model)

##### Computation of degrees of freedom (Default model)

|  |  |
| --- | --- |
| Number of distinct sample moments: | 253 |
| Number of distinct parameters to be estimated: | 50 |
| Degrees of freedom (253 - 50): | 203 |

##### Result (Default model)

Minimum was achieved

Chi-square = 823.781

Degrees of freedom = 203

Probability level = .000

##### Group number 1 (Group number 1 - Default model)

##### Estimates (Group number 1 - Default model)

##### Scalar Estimates (Group number 1 - Default model)

##### Maximum Likelihood Estimates

##### Regression Weights: (Group number 1 - Default model)

|  |  |  | Estimate | S.E. | C.R. | P | Label |
| --- | --- | --- | --- | --- | --- | --- | --- |
| y12 | <--- | F1 | 1.000 |  |
| y11 | <--- | F1 | 1.037 | .075 | 13.794 | \*\*\* |  |
| y10 | <--- | F1 | 1.021 | .079 | 12.860 | \*\*\* |  |
| y9 | <--- | F1 | .776 | .084 | 9.284 | \*\*\* |  |
| y8 | <--- | F1 | .836 | .091 | 9.224 | \*\*\* |  |
| y7 | <--- | F1 | .792 | .088 | 9.033 | \*\*\* |  |
| y18 | <--- | F2 | 1.000 |  |
| y17 | <--- | F2 | .599 | .079 | 7.590 | \*\*\* |  |
| y16 | <--- | F2 | 1.006 | .075 | 13.492 | \*\*\* |  |
| y15 | <--- | F2 | .910 | .074 | 12.300 | \*\*\* |  |
| y14 | <--- | F2 | .789 | .063 | 12.593 | \*\*\* |  |
| y13 | <--- | F2 | .666 | .062 | 10.790 | \*\*\* |  |
| y6 | <--- | F3 | 1.000 |  |
| y5 | <--- | F3 | 1.111 | .072 | 15.332 | \*\*\* |  |
| y4 | <--- | F3 | .795 | .061 | 13.110 | \*\*\* |  |
| y3 | <--- | F3 | .875 | .076 | 11.518 | \*\*\* |  |
| y2 | <--- | F3 | .779 | .080 | 9.701 | \*\*\* |  |
| y1 | <--- | F3 | .702 | .080 | 8.796 | \*\*\* |  |
| y22 | <--- | F4 | 1.000 |  |
| y21 | <--- | F4 | .713 | .086 | 8.277 | \*\*\* |  |
| y20 | <--- | F4 | .934 | .092 | 10.150 | \*\*\* |  |
| y19 | <--- | F4 | .986 | .098 | 10.097 | \*\*\* |  |

##### Standardized Regression Weights: (Group number 1 - Default model)

|  |  |  | Estimate |
| --- | --- | --- | --- |
| y12 | <--- | F1 | .786 |
| y11 | <--- | F1 | .837 |
| y10 | <--- | F1 | .785 |
| y9 | <--- | F1 | .590 |
| y8 | <--- | F1 | .586 |
| y7 | <--- | F1 | .575 |
| y18 | <--- | F2 | .807 |
| y17 | <--- | F2 | .486 |
| y16 | <--- | F2 | .798 |
| y15 | <--- | F2 | .739 |
| y14 | <--- | F2 | .754 |
| y13 | <--- | F2 | .663 |
| y6 | <--- | F3 | .843 |
| y5 | <--- | F3 | .844 |
| y4 | <--- | F3 | .747 |
| y3 | <--- | F3 | .677 |
| y2 | <--- | F3 | .590 |
| y1 | <--- | F3 | .543 |
| y22 | <--- | F4 | .735 |
| y21 | <--- | F4 | .591 |
| y20 | <--- | F4 | .752 |
| y19 | <--- | F4 | .746 |

##### Covariances: (Group number 1 - Default model)

|  |  |  | Estimate | S.E. | C.R. | P | Label |
| --- | --- | --- | --- | --- | --- | --- | --- |
| F1 | <--> | F2 | -.344 | .055 | -6.267 | \*\*\* |  |
| F1 | <--> | F3 | .207 | .055 | 3.783 | \*\*\* |  |
| F1 | <--> | F4 | .277 | .055 | 5.010 | \*\*\* |  |
| F2 | <--> | F3 | -.178 | .054 | -3.281 | .001 |  |
| F2 | <--> | F4 | -.132 | .050 | -2.648 | .008 |  |
| F3 | <--> | F4 | .072 | .056 | 1.303 | .193 |  |

##### Correlations: (Group number 1 - Default model)

|  |  |  | Estimate |
| --- | --- | --- | --- |
| F1 | <--> | F2 | -.549 |
| F1 | <--> | F3 | .287 |
| F1 | <--> | F4 | .433 |
| F2 | <--> | F3 | -.244 |
| F2 | <--> | F4 | -.205 |
| F3 | <--> | F4 | .098 |

##### Variances: (Group number 1 - Default model)

|  |  |  | Estimate | S.E. | C.R. | P | Label |
| --- | --- | --- | --- | --- | --- | --- | --- |
| F1 |  |  | .622 | .087 | 7.117 | \*\*\* |  |
| F2 |  |  | .630 | .085 | 7.397 | \*\*\* |  |
| F3 |  |  | .838 | .106 | 7.916 | \*\*\* |  |
| F4 |  |  | .657 | .108 | 6.105 | \*\*\* |  |
| e1 |  |  | .385 | .044 | 8.783 | \*\*\* |  |
| e2 |  |  | .285 | .037 | 7.695 | \*\*\* |  |
| e3 |  |  | .405 | .046 | 8.802 | \*\*\* |  |
| e4 |  |  | .703 | .067 | 10.422 | \*\*\* |  |
| e5 |  |  | .831 | .080 | 10.436 | \*\*\* |  |
| e6 |  |  | .789 | .075 | 10.479 | \*\*\* |  |
| e7 |  |  | .337 | .040 | 8.378 | \*\*\* |  |
| e8 |  |  | .730 | .068 | 10.745 | \*\*\* |  |
| e9 |  |  | .362 | .042 | 8.548 | \*\*\* |  |
| e10 |  |  | .433 | .046 | 9.395 | \*\*\* |  |
| e11 |  |  | .298 | .032 | 9.225 | \*\*\* |  |
| e12 |  |  | .357 | .036 | 10.036 | \*\*\* |  |
| e13 |  |  | .340 | .045 | 7.616 | \*\*\* |  |
| e14 |  |  | .420 | .055 | 7.611 | \*\*\* |  |
| e15 |  |  | .418 | .045 | 9.396 | \*\*\* |  |
| e16 |  |  | .759 | .076 | 10.007 | \*\*\* |  |
| e17 |  |  | .955 | .091 | 10.455 | \*\*\* |  |
| e18 |  |  | .987 | .093 | 10.618 | \*\*\* |  |
| e19 |  |  | .560 | .069 | 8.103 | \*\*\* |  |
| e20 |  |  | .625 | .064 | 9.826 | \*\*\* |  |
| e21 |  |  | .441 | .057 | 7.758 | \*\*\* |  |
| e22 |  |  | .510 | .065 | 7.889 | \*\*\* |  |

##### Modification Indices (Group number 1 - Default model)

##### Covariances: (Group number 1 - Default model)

|  |  |  | M.I. | Par Change |
| --- | --- | --- | --- | --- |
| e20 | <--> | F2 | 5.657 | .092 |
| e19 | <--> | F1 | 12.876 | .131 |
| e18 | <--> | e20 | 4.668 | .115 |
| e17 | <--> | e18 | 98.651 | .642 |
| e16 | <--> | e18 | 43.174 | .385 |
| e16 | <--> | e17 | 24.736 | .288 |
| e15 | <--> | e18 | 15.136 | -.173 |
| e15 | <--> | e17 | 4.669 | -.095 |
| e14 | <--> | e22 | 4.229 | -.080 |
| e14 | <--> | e18 | 10.160 | -.154 |
| e14 | <--> | e17 | 12.642 | -.170 |
| e14 | <--> | e16 | 6.049 | -.106 |
| e14 | <--> | e15 | 5.655 | .077 |
| e13 | <--> | e22 | 4.382 | .073 |
| e13 | <--> | e18 | 11.948 | -.150 |
| e13 | <--> | e17 | 9.020 | -.129 |
| e13 | <--> | e14 | 10.655 | .100 |
| e12 | <--> | F4 | 8.734 | .098 |
| e12 | <--> | F1 | 4.595 | .058 |
| e12 | <--> | e20 | 4.793 | .072 |
| e11 | <--> | F1 | 4.734 | .056 |
| e11 | <--> | e15 | 5.317 | -.060 |
| e11 | <--> | e13 | 10.856 | .084 |
| e11 | <--> | e12 | 65.926 | .189 |
| e10 | <--> | F3 | 5.016 | -.094 |
| e10 | <--> | e12 | 16.072 | -.112 |
| e9 | <--> | e12 | 12.126 | -.092 |
| e9 | <--> | e10 | 15.131 | .115 |
| e8 | <--> | e19 | 8.885 | -.138 |
| e8 | <--> | e12 | 5.578 | .081 |
| e8 | <--> | e10 | 9.717 | -.121 |
| e7 | <--> | F1 | 6.310 | -.071 |
| e7 | <--> | e14 | 6.970 | .083 |
| e7 | <--> | e11 | 6.096 | -.059 |
| e6 | <--> | F2 | 18.057 | .181 |
| e6 | <--> | e20 | 5.992 | .118 |
| e5 | <--> | F2 | 15.727 | .173 |
| e5 | <--> | e12 | 5.543 | .088 |
| e5 | <--> | e8 | 4.505 | .110 |
| e5 | <--> | e6 | 110.784 | .570 |
| e4 | <--> | e20 | 5.025 | .102 |
| e4 | <--> | e16 | 4.766 | -.109 |
| e4 | <--> | e6 | 52.819 | .362 |
| e4 | <--> | e5 | 67.617 | .422 |
| e3 | <--> | e20 | 6.695 | -.096 |
| e3 | <--> | e14 | 4.180 | -.069 |
| e2 | <--> | e6 | 10.477 | -.115 |
| e2 | <--> | e5 | 11.904 | -.126 |
| e2 | <--> | e4 | 6.810 | -.088 |
| e1 | <--> | F2 | 19.962 | -.142 |
| e1 | <--> | F1 | 4.743 | -.063 |
| e1 | <--> | e9 | 5.787 | -.070 |
| e1 | <--> | e6 | 17.510 | -.165 |
| e1 | <--> | e5 | 19.722 | -.180 |
| e1 | <--> | e4 | 20.195 | -.168 |
| e1 | <--> | e2 | 12.849 | .093 |

##### Variances: (Group number 1 - Default model)

|  |  |  | M.I. | Par Change |
| --- | --- | --- | --- | --- |

##### Regression Weights: (Group number 1 - Default model)

|  |  |  | M.I. | Par Change |
| --- | --- | --- | --- | --- |
| y21 | <--- | F2 | 14.209 | .265 |
| y21 | <--- | F1 | 10.537 | -.230 |
| y21 | <--- | y13 | 15.306 | .258 |
| y21 | <--- | y14 | 13.672 | .234 |
| y21 | <--- | y15 | 4.003 | .108 |
| y21 | <--- | y16 | 4.085 | .106 |
| y21 | <--- | y17 | 7.211 | .144 |
| y21 | <--- | y18 | 11.125 | .178 |
| y21 | <--- | y10 | 14.792 | -.197 |
| y21 | <--- | y11 | 9.009 | -.161 |
| y21 | <--- | y12 | 12.235 | -.183 |
| y22 | <--- | F2 | 9.943 | -.225 |
| y22 | <--- | F1 | 16.427 | .291 |
| y22 | <--- | y14 | 5.149 | -.146 |
| y22 | <--- | y15 | 5.978 | -.133 |
| y22 | <--- | y17 | 15.993 | -.218 |
| y22 | <--- | y18 | 8.477 | -.158 |
| y22 | <--- | y10 | 16.357 | .210 |
| y22 | <--- | y11 | 15.919 | .218 |
| y22 | <--- | y12 | 14.983 | .206 |
| y1 | <--- | y2 | 60.758 | .414 |
| y1 | <--- | y3 | 21.310 | .250 |
| y1 | <--- | y4 | 5.796 | -.159 |
| y2 | <--- | y1 | 66.558 | .438 |
| y2 | <--- | y3 | 12.227 | .188 |
| y3 | <--- | y1 | 29.185 | .264 |
| y3 | <--- | y2 | 15.286 | .187 |
| y3 | <--- | y9 | 5.227 | -.127 |
| y4 | <--- | y1 | 10.261 | -.119 |
| y4 | <--- | y14 | 7.045 | -.140 |
| y5 | <--- | y1 | 6.957 | -.107 |
| y5 | <--- | y2 | 7.943 | -.112 |
| y5 | <--- | y18 | 7.061 | .129 |
| y6 | <--- | y19 | 4.144 | .082 |
| y6 | <--- | y1 | 8.181 | -.104 |
| y6 | <--- | y2 | 5.667 | -.085 |
| y6 | <--- | y14 | 6.250 | .130 |
| y13 | <--- | F4 | 15.525 | .213 |
| y13 | <--- | F1 | 8.527 | .156 |
| y13 | <--- | y19 | 13.941 | .138 |
| y13 | <--- | y21 | 14.296 | .153 |
| y13 | <--- | y22 | 12.092 | .125 |
| y13 | <--- | y14 | 24.553 | .236 |
| y13 | <--- | y15 | 6.371 | -.102 |
| y13 | <--- | y17 | 4.122 | .082 |
| y13 | <--- | y7 | 9.112 | .110 |
| y13 | <--- | y8 | 11.973 | .122 |
| y13 | <--- | y12 | 7.190 | .106 |
| y14 | <--- | F4 | 6.320 | .129 |
| y14 | <--- | F1 | 6.258 | .126 |
| y14 | <--- | y19 | 8.012 | .099 |
| y14 | <--- | y21 | 6.979 | .101 |
| y14 | <--- | y6 | 6.490 | .088 |
| y14 | <--- | y13 | 34.213 | .275 |
| y14 | <--- | y7 | 8.226 | .099 |
| y14 | <--- | y8 | 5.435 | .078 |
| y14 | <--- | y10 | 5.758 | .088 |
| y15 | <--- | F3 | 5.218 | -.118 |
| y15 | <--- | y19 | 4.170 | -.085 |
| y15 | <--- | y1 | 4.274 | -.078 |
| y15 | <--- | y6 | 6.889 | -.108 |
| y15 | <--- | y13 | 8.326 | -.162 |
| y15 | <--- | y16 | 4.548 | .096 |
| y15 | <--- | y17 | 7.196 | -.123 |
| y16 | <--- | y19 | 4.052 | -.080 |
| y16 | <--- | y13 | 6.340 | -.134 |
| y16 | <--- | y15 | 6.148 | .108 |
| y16 | <--- | y12 | 5.964 | -.104 |
| y17 | <--- | y22 | 4.241 | -.103 |
| y18 | <--- | F1 | 4.922 | -.124 |
| y18 | <--- | y7 | 4.401 | -.080 |
| y18 | <--- | y10 | 4.229 | -.083 |
| y18 | <--- | y12 | 4.865 | -.091 |
| y7 | <--- | F2 | 11.144 | .258 |
| y7 | <--- | y21 | 4.097 | .119 |
| y7 | <--- | y13 | 11.375 | .244 |
| y7 | <--- | y14 | 12.381 | .244 |
| y7 | <--- | y15 | 5.337 | .136 |
| y7 | <--- | y16 | 9.747 | .180 |
| y7 | <--- | y17 | 10.243 | .189 |
| y7 | <--- | y8 | 68.614 | .425 |
| y7 | <--- | y9 | 32.477 | .317 |
| y7 | <--- | y12 | 5.528 | -.135 |
| y8 | <--- | F2 | 9.835 | .249 |
| y8 | <--- | y13 | 13.217 | .270 |
| y8 | <--- | y14 | 8.734 | .211 |
| y8 | <--- | y16 | 7.766 | .165 |
| y8 | <--- | y17 | 10.595 | .198 |
| y8 | <--- | y18 | 4.986 | .135 |
| y8 | <--- | y7 | 70.194 | .458 |
| y8 | <--- | y9 | 41.586 | .369 |
| y8 | <--- | y12 | 6.232 | -.148 |
| y9 | <--- | y7 | 33.469 | .291 |
| y9 | <--- | y8 | 41.892 | .314 |
| y9 | <--- | y12 | 6.383 | -.137 |
| y10 | <--- | y21 | 6.628 | -.117 |
| y11 | <--- | y7 | 6.759 | -.094 |
| y11 | <--- | y8 | 7.517 | -.096 |
| y11 | <--- | y9 | 4.270 | -.078 |
| y11 | <--- | y12 | 4.336 | .082 |
| y12 | <--- | F2 | 11.805 | -.200 |
| y12 | <--- | y14 | 5.866 | -.126 |
| y12 | <--- | y15 | 9.038 | -.133 |
| y12 | <--- | y16 | 14.995 | -.168 |
| y12 | <--- | y17 | 6.036 | -.109 |
| y12 | <--- | y18 | 9.840 | -.138 |
| y12 | <--- | y7 | 11.203 | -.134 |
| y12 | <--- | y8 | 12.345 | -.135 |
| y12 | <--- | y9 | 12.552 | -.148 |

##### Bootstrap (Group number 1 - Default model)

##### Bootstrap standard errors (Group number 1 - Default model)

##### Scalar Estimates (Group number 1 - Default model)

##### Regression Weights: (Group number 1 - Default model)

| Parameter | | | SE | SE-SE | Mean | Bias | SE-Bias |
| --- | --- | --- | --- | --- | --- | --- | --- |
| y12 | <--- | F1 | .000 | .000 | 1.000 | .000 | .000 |
| y11 | <--- | F1 | .080 | .001 | 1.048 | .011 | .001 |
| y10 | <--- | F1 | .120 | .001 | 1.040 | .019 | .002 |
| y9 | <--- | F1 | .282 | .003 | .840 | .064 | .004 |
| y8 | <--- | F1 | .344 | .003 | .920 | .084 | .005 |
| y7 | <--- | F1 | .341 | .003 | .870 | .078 | .005 |
| y18 | <--- | F2 | .000 | .000 | 1.000 | .000 | .000 |
| y17 | <--- | F2 | .099 | .001 | .600 | .001 | .001 |
| y16 | <--- | F2 | .092 | .001 | 1.008 | .002 | .001 |
| y15 | <--- | F2 | .090 | .001 | .911 | .001 | .001 |
| y14 | <--- | F2 | .099 | .001 | .798 | .009 | .001 |
| y13 | <--- | F2 | .097 | .001 | .672 | .006 | .001 |
| y6 | <--- | F3 | .000 | .000 | 1.000 | .000 | .000 |
| y5 | <--- | F3 | .053 | .001 | 1.114 | .002 | .001 |
| y4 | <--- | F3 | .071 | .001 | .794 | .000 | .001 |
| y3 | <--- | F3 | .110 | .001 | .888 | .013 | .002 |
| y2 | <--- | F3 | .142 | .001 | .797 | .018 | .002 |
| y1 | <--- | F3 | .147 | .001 | .720 | .018 | .002 |
| y22 | <--- | F4 | .000 | .000 | 1.000 | .000 | .000 |
| y21 | <--- | F4 | .107 | .001 | .719 | .005 | .002 |
| y20 | <--- | F4 | .120 | .001 | .942 | .007 | .002 |
| y19 | <--- | F4 | .119 | .001 | .995 | .009 | .002 |

##### Standardized Regression Weights: (Group number 1 - Default model)

| Parameter | | | SE | SE-SE | Mean | Bias | SE-Bias |
| --- | --- | --- | --- | --- | --- | --- | --- |
| y12 | <--- | F1 | .090 | .001 | .767 | -.019 | .001 |
| y11 | <--- | F1 | .079 | .001 | .821 | -.017 | .001 |
| y10 | <--- | F1 | .063 | .001 | .771 | -.014 | .001 |
| y9 | <--- | F1 | .087 | .001 | .598 | .008 | .001 |
| y8 | <--- | F1 | .103 | .001 | .601 | .015 | .001 |
| y7 | <--- | F1 | .107 | .001 | .588 | .012 | .002 |
| y18 | <--- | F2 | .035 | .000 | .805 | -.003 | .000 |
| y17 | <--- | F2 | .072 | .001 | .484 | -.002 | .001 |
| y16 | <--- | F2 | .051 | .001 | .795 | -.003 | .001 |
| y15 | <--- | F2 | .064 | .001 | .737 | -.002 | .001 |
| y14 | <--- | F2 | .051 | .001 | .756 | .002 | .001 |
| y13 | <--- | F2 | .067 | .001 | .664 | .001 | .001 |
| y6 | <--- | F3 | .042 | .000 | .838 | -.005 | .001 |
| y5 | <--- | F3 | .043 | .000 | .838 | -.006 | .001 |
| y4 | <--- | F3 | .056 | .001 | .741 | -.006 | .001 |
| y3 | <--- | F3 | .045 | .000 | .677 | .000 | .001 |
| y2 | <--- | F3 | .059 | .001 | .592 | .003 | .001 |
| y1 | <--- | F3 | .067 | .001 | .547 | .004 | .001 |
| y22 | <--- | F4 | .059 | .001 | .734 | .000 | .001 |
| y21 | <--- | F4 | .059 | .001 | .590 | -.001 | .001 |
| y20 | <--- | F4 | .047 | .000 | .751 | -.001 | .001 |
| y19 | <--- | F4 | .040 | .000 | .745 | .000 | .001 |

##### Covariances: (Group number 1 - Default model)

| Parameter | | | SE | SE-SE | Mean | Bias | SE-Bias |
| --- | --- | --- | --- | --- | --- | --- | --- |
| F1 | <--> | F2 | .095 | .001 | -.328 | .016 | .001 |
| F1 | <--> | F3 | .070 | .001 | .198 | -.009 | .001 |
| F1 | <--> | F4 | .088 | .001 | .270 | -.007 | .001 |
| F2 | <--> | F3 | .053 | .001 | -.172 | .005 | .001 |
| F2 | <--> | F4 | .065 | .001 | -.130 | .002 | .001 |
| F3 | <--> | F4 | .055 | .001 | .070 | -.003 | .001 |

##### Correlations: (Group number 1 - Default model)

| Parameter | | | SE | SE-SE | Mean | Bias | SE-Bias |
| --- | --- | --- | --- | --- | --- | --- | --- |
| F1 | <--> | F2 | .102 | .001 | -.529 | .020 | .001 |
| F1 | <--> | F3 | .081 | .001 | .281 | -.006 | .001 |
| F1 | <--> | F4 | .084 | .001 | .424 | -.009 | .001 |
| F2 | <--> | F3 | .069 | .001 | -.241 | .003 | .001 |
| F2 | <--> | F4 | .090 | .001 | -.200 | .005 | .001 |
| F3 | <--> | F4 | .072 | .001 | .094 | -.003 | .001 |

##### Variances: (Group number 1 - Default model)

| Parameter | | | SE | SE-SE | Mean | Bias | SE-Bias |
| --- | --- | --- | --- | --- | --- | --- | --- |
| F1 |  |  | .145 | .001 | .600 | -.023 | .002 |
| F2 |  |  | .077 | .001 | .624 | -.006 | .001 |
| F3 |  |  | .139 | .001 | .829 | -.009 | .002 |
| F4 |  |  | .122 | .001 | .659 | .002 | .002 |
| e1 |  |  | .115 | .001 | .402 | .017 | .002 |
| e2 |  |  | .111 | .001 | .303 | .018 | .002 |
| e3 |  |  | .095 | .001 | .419 | .014 | .001 |
| e4 |  |  | .115 | .001 | .680 | -.024 | .002 |
| e5 |  |  | .167 | .002 | .790 | -.041 | .002 |
| e6 |  |  | .159 | .002 | .753 | -.037 | .002 |
| e7 |  |  | .058 | .001 | .337 | .000 | .001 |
| e8 |  |  | .107 | .001 | .725 | -.005 | .002 |
| e9 |  |  | .084 | .001 | .362 | .000 | .001 |
| e10 |  |  | .101 | .001 | .431 | -.002 | .001 |
| e11 |  |  | .048 | .000 | .290 | -.008 | .001 |
| e12 |  |  | .055 | .001 | .348 | -.009 | .001 |
| e13 |  |  | .072 | .001 | .344 | .004 | .001 |
| e14 |  |  | .086 | .001 | .427 | .008 | .001 |
| e15 |  |  | .051 | .001 | .415 | -.003 | .001 |
| e16 |  |  | .083 | .001 | .751 | -.008 | .001 |
| e17 |  |  | .105 | .001 | .939 | -.017 | .001 |
| e18 |  |  | .113 | .001 | .969 | -.018 | .002 |
| e19 |  |  | .102 | .001 | .552 | -.008 | .001 |
| e20 |  |  | .080 | .001 | .619 | -.006 | .001 |
| e21 |  |  | .071 | .001 | .438 | -.003 | .001 |
| e22 |  |  | .066 | .001 | .506 | -.005 | .001 |

##### Bootstrap Confidence (Group number 1 - Default model)

##### Percentile method (Group number 1 - Default model)

##### 95% confidence intervals (percentile method)

##### Scalar Estimates (Group number 1 - Default model)

##### Regression Weights: (Group number 1 - Default model)

| Parameter | | | Estimate | Lower | Upper | P |
| --- | --- | --- | --- | --- | --- | --- |
| y12 | <--- | F1 | 1.000 | 1.000 | 1.000 | ... |
| y11 | <--- | F1 | 1.037 | .914 | 1.227 | .000 |
| y10 | <--- | F1 | 1.021 | .852 | 1.327 | .000 |
| y9 | <--- | F1 | .776 | .546 | 1.726 | .000 |
| y8 | <--- | F1 | .836 | .583 | 2.042 | .000 |
| y7 | <--- | F1 | .792 | .528 | 1.960 | .000 |
| y18 | <--- | F2 | 1.000 | 1.000 | 1.000 | ... |
| y17 | <--- | F2 | .599 | .405 | .797 | .000 |
| y16 | <--- | F2 | 1.006 | .839 | 1.198 | .000 |
| y15 | <--- | F2 | .910 | .744 | 1.098 | .000 |
| y14 | <--- | F2 | .789 | .613 | .996 | .000 |
| y13 | <--- | F2 | .666 | .500 | .880 | .000 |
| y6 | <--- | F3 | 1.000 | 1.000 | 1.000 | ... |
| y5 | <--- | F3 | 1.111 | 1.017 | 1.227 | .000 |
| y4 | <--- | F3 | .795 | .650 | .927 | .000 |
| y3 | <--- | F3 | .875 | .730 | 1.123 | .000 |
| y2 | <--- | F3 | .779 | .609 | 1.080 | .000 |
| y1 | <--- | F3 | .702 | .528 | 1.006 | .000 |
| y22 | <--- | F4 | 1.000 | 1.000 | 1.000 | ... |
| y21 | <--- | F4 | .713 | .523 | .947 | .000 |
| y20 | <--- | F4 | .934 | .730 | 1.207 | .000 |
| y19 | <--- | F4 | .986 | .790 | 1.260 | .000 |

##### Standardized Regression Weights: (Group number 1 - Default model)

| Parameter | | | Estimate | Lower | Upper | P |
| --- | --- | --- | --- | --- | --- | --- |
| y12 | <--- | F1 | .786 | .488 | .872 | .000 |
| y11 | <--- | F1 | .837 | .574 | .911 | .000 |
| y10 | <--- | F1 | .785 | .591 | .858 | .000 |
| y9 | <--- | F1 | .590 | .445 | .796 | .000 |
| y8 | <--- | F1 | .586 | .432 | .860 | .000 |
| y7 | <--- | F1 | .575 | .416 | .858 | .000 |
| y18 | <--- | F2 | .807 | .729 | .868 | .000 |
| y17 | <--- | F2 | .486 | .338 | .620 | .000 |
| y16 | <--- | F2 | .798 | .681 | .880 | .000 |
| y15 | <--- | F2 | .739 | .598 | .844 | .000 |
| y14 | <--- | F2 | .754 | .649 | .849 | .000 |
| y13 | <--- | F2 | .663 | .530 | .796 | .000 |
| y6 | <--- | F3 | .843 | .747 | .893 | .000 |
| y5 | <--- | F3 | .844 | .750 | .895 | .000 |
| y4 | <--- | F3 | .747 | .619 | .827 | .000 |
| y3 | <--- | F3 | .677 | .586 | .763 | .000 |
| y2 | <--- | F3 | .590 | .481 | .714 | .000 |
| y1 | <--- | F3 | .543 | .423 | .685 | .000 |
| y22 | <--- | F4 | .735 | .612 | .840 | .000 |
| y21 | <--- | F4 | .591 | .468 | .700 | .000 |
| y20 | <--- | F4 | .752 | .648 | .833 | .000 |
| y19 | <--- | F4 | .746 | .661 | .819 | .000 |

##### Covariances: (Group number 1 - Default model)

| Parameter | | | Estimate | Lower | Upper | P |
| --- | --- | --- | --- | --- | --- | --- |
| F1 | <--> | F2 | -.344 | -.485 | -.088 | .000 |
| F1 | <--> | F3 | .207 | .067 | .339 | .001 |
| F1 | <--> | F4 | .277 | .096 | .446 | .000 |
| F2 | <--> | F3 | -.178 | -.278 | -.068 | .002 |
| F2 | <--> | F4 | -.132 | -.262 | -.010 | .033 |
| F3 | <--> | F4 | .072 | -.034 | .183 | .190 |

##### Correlations: (Group number 1 - Default model)

| Parameter | | | Estimate | Lower | Upper | P |
| --- | --- | --- | --- | --- | --- | --- |
| F1 | <--> | F2 | -.549 | -.675 | -.244 | .000 |
| F1 | <--> | F3 | .287 | .124 | .438 | .001 |
| F1 | <--> | F4 | .433 | .247 | .577 | .000 |
| F2 | <--> | F3 | -.244 | -.376 | -.101 | .002 |
| F2 | <--> | F4 | -.205 | -.372 | -.019 | .033 |
| F3 | <--> | F4 | .098 | -.048 | .234 | .190 |

##### Variances: (Group number 1 - Default model)

| Parameter | | | Estimate | Lower | Upper | P |
| --- | --- | --- | --- | --- | --- | --- |
| F1 |  |  | .622 | .220 | .833 | .000 |
| F2 |  |  | .630 | .477 | .782 | .000 |
| F3 |  |  | .838 | .560 | 1.087 | .000 |
| F4 |  |  | .657 | .427 | .904 | .000 |
| e1 |  |  | .385 | .241 | .721 | .000 |
| e2 |  |  | .285 | .158 | .608 | .000 |
| e3 |  |  | .405 | .272 | .668 | .000 |
| e4 |  |  | .703 | .398 | .864 | .000 |
| e5 |  |  | .831 | .321 | 1.022 | .000 |
| e6 |  |  | .789 | .308 | .977 | .000 |
| e7 |  |  | .337 | .229 | .458 | .000 |
| e8 |  |  | .730 | .522 | .943 | .000 |
| e9 |  |  | .362 | .217 | .552 | .000 |
| e10 |  |  | .433 | .258 | .655 | .000 |
| e11 |  |  | .298 | .192 | .381 | .000 |
| e12 |  |  | .357 | .236 | .454 | .000 |
| e13 |  |  | .340 | .228 | .503 | .000 |
| e14 |  |  | .420 | .291 | .617 | .000 |
| e15 |  |  | .418 | .321 | .526 | .000 |
| e16 |  |  | .759 | .590 | .912 | .000 |
| e17 |  |  | .955 | .712 | 1.126 | .000 |
| e18 |  |  | .987 | .739 | 1.170 | .000 |
| e19 |  |  | .560 | .355 | .762 | .000 |
| e20 |  |  | .625 | .470 | .786 | .000 |
| e21 |  |  | .441 | .308 | .586 | .000 |
| e22 |  |  | .510 | .375 | .639 | .000 |

##### Bias-corrected percentile method (Group number 1 - Default model)

##### 95% confidence intervals (bias-corrected percentile method)

##### Scalar Estimates (Group number 1 - Default model)

##### Regression Weights: (Group number 1 - Default model)

| Parameter | | | Estimate | Lower | Upper | P |
| --- | --- | --- | --- | --- | --- | --- |
| y12 | <--- | F1 | 1.000 | 1.000 | 1.000 | ... |
| y11 | <--- | F1 | 1.037 | .906 | 1.217 | .001 |
| y10 | <--- | F1 | 1.021 | .841 | 1.305 | .001 |
| y9 | <--- | F1 | .776 | .554 | 1.776 | .000 |
| y8 | <--- | F1 | .836 | .590 | 2.090 | .000 |
| y7 | <--- | F1 | .792 | .537 | 2.027 | .000 |
| y18 | <--- | F2 | 1.000 | 1.000 | 1.000 | ... |
| y17 | <--- | F2 | .599 | .406 | .798 | .000 |
| y16 | <--- | F2 | 1.006 | .842 | 1.201 | .000 |
| y15 | <--- | F2 | .910 | .749 | 1.107 | .000 |
| y14 | <--- | F2 | .789 | .604 | .986 | .001 |
| y13 | <--- | F2 | .666 | .504 | .883 | .000 |
| y6 | <--- | F3 | 1.000 | 1.000 | 1.000 | ... |
| y5 | <--- | F3 | 1.111 | 1.018 | 1.228 | .000 |
| y4 | <--- | F3 | .795 | .641 | .922 | .001 |
| y3 | <--- | F3 | .875 | .734 | 1.149 | .000 |
| y2 | <--- | F3 | .779 | .612 | 1.102 | .000 |
| y1 | <--- | F3 | .702 | .532 | 1.038 | .000 |
| y22 | <--- | F4 | 1.000 | 1.000 | 1.000 | ... |
| y21 | <--- | F4 | .713 | .524 | .950 | .000 |
| y20 | <--- | F4 | .934 | .733 | 1.211 | .000 |
| y19 | <--- | F4 | .986 | .796 | 1.264 | .000 |

##### Standardized Regression Weights: (Group number 1 - Default model)

| Parameter | | | Estimate | Lower | Upper | P |
| --- | --- | --- | --- | --- | --- | --- |
| y12 | <--- | F1 | .786 | .477 | .870 | .001 |
| y11 | <--- | F1 | .837 | .562 | .908 | .001 |
| y10 | <--- | F1 | .785 | .607 | .861 | .000 |
| y9 | <--- | F1 | .590 | .445 | .796 | .000 |
| y8 | <--- | F1 | .586 | .429 | .857 | .000 |
| y7 | <--- | F1 | .575 | .418 | .860 | .000 |
| y18 | <--- | F2 | .807 | .730 | .869 | .000 |
| y17 | <--- | F2 | .486 | .338 | .620 | .000 |
| y16 | <--- | F2 | .798 | .673 | .877 | .001 |
| y15 | <--- | F2 | .739 | .590 | .840 | .001 |
| y14 | <--- | F2 | .754 | .641 | .843 | .001 |
| y13 | <--- | F2 | .663 | .527 | .793 | .000 |
| y6 | <--- | F3 | .843 | .731 | .891 | .001 |
| y5 | <--- | F3 | .844 | .751 | .895 | .000 |
| y4 | <--- | F3 | .747 | .620 | .827 | .000 |
| y3 | <--- | F3 | .677 | .581 | .759 | .001 |
| y2 | <--- | F3 | .590 | .479 | .708 | .000 |
| y1 | <--- | F3 | .543 | .419 | .680 | .000 |
| y22 | <--- | F4 | .735 | .604 | .834 | .001 |
| y21 | <--- | F4 | .591 | .465 | .699 | .000 |
| y20 | <--- | F4 | .752 | .644 | .831 | .000 |
| y19 | <--- | F4 | .746 | .659 | .818 | .001 |

##### Covariances: (Group number 1 - Default model)

| Parameter | | | Estimate | Lower | Upper | P |
| --- | --- | --- | --- | --- | --- | --- |
| F1 | <--> | F2 | -.344 | -.496 | -.104 | .000 |
| F1 | <--> | F3 | .207 | .085 | .359 | .000 |
| F1 | <--> | F4 | .277 | .113 | .463 | .000 |
| F2 | <--> | F3 | -.178 | -.289 | -.080 | .001 |
| F2 | <--> | F4 | -.132 | -.272 | -.015 | .025 |
| F3 | <--> | F4 | .072 | -.025 | .196 | .134 |

##### Correlations: (Group number 1 - Default model)

| Parameter | | | Estimate | Lower | Upper | P |
| --- | --- | --- | --- | --- | --- | --- |
| F1 | <--> | F2 | -.549 | -.676 | -.251 | .000 |
| F1 | <--> | F3 | .287 | .132 | .445 | .001 |
| F1 | <--> | F4 | .433 | .257 | .586 | .000 |
| F2 | <--> | F3 | -.244 | -.380 | -.104 | .001 |
| F2 | <--> | F4 | -.205 | -.376 | -.024 | .028 |
| F3 | <--> | F4 | .098 | -.040 | .244 | .151 |

##### Variances: (Group number 1 - Default model)

| Parameter | | | Estimate | Lower | Upper | P |
| --- | --- | --- | --- | --- | --- | --- |
| F1 |  |  | .622 | .232 | .842 | .000 |
| F2 |  |  | .630 | .490 | .796 | .000 |
| F3 |  |  | .838 | .569 | 1.095 | .000 |
| F4 |  |  | .657 | .424 | .902 | .000 |
| e1 |  |  | .385 | .248 | .735 | .000 |
| e2 |  |  | .285 | .166 | .630 | .000 |
| e3 |  |  | .405 | .271 | .667 | .000 |
| e4 |  |  | .703 | .415 | .877 | .000 |
| e5 |  |  | .831 | .355 | 1.039 | .000 |
| e6 |  |  | .789 | .327 | .984 | .000 |
| e7 |  |  | .337 | .232 | .462 | .000 |
| e8 |  |  | .730 | .540 | .965 | .000 |
| e9 |  |  | .362 | .228 | .570 | .000 |
| e10 |  |  | .433 | .277 | .685 | .000 |
| e11 |  |  | .298 | .212 | .396 | .000 |
| e12 |  |  | .357 | .257 | .472 | .000 |
| e13 |  |  | .340 | .236 | .533 | .000 |
| e14 |  |  | .420 | .292 | .620 | .000 |
| e15 |  |  | .418 | .335 | .544 | .000 |
| e16 |  |  | .759 | .608 | .930 | .000 |
| e17 |  |  | .955 | .762 | 1.148 | .000 |
| e18 |  |  | .987 | .784 | 1.202 | .000 |
| e19 |  |  | .560 | .374 | .778 | .000 |
| e20 |  |  | .625 | .485 | .807 | .000 |
| e21 |  |  | .441 | .321 | .603 | .000 |
| e22 |  |  | .510 | .385 | .649 | .000 |

##### Minimization History (Default model)

| Iteration |  | Negative eigenvalues | Condition # | Smallest eigenvalue | Diameter | F | NTries | Ratio |
| --- | --- | --- | --- | --- | --- | --- | --- | --- |
| 0 | e | 9 |  | -.692 | 9999.000 | 2990.716 | 0 | 9999.000 |
| 1 | e | 8 |  | -.228 | 4.024 | 1381.816 | 20 | .306 |
| 2 | e | 4 |  | -.056 | .662 | 1083.755 | 6 | .896 |
| 3 | e | 0 | 1143.754 |  | 1.038 | 898.909 | 6 | .668 |
| 4 | e | 0 | 104.939 |  | .742 | 863.439 | 5 | .000 |
| 5 | e | 0 | 70.563 |  | .754 | 833.803 | 1 | .785 |
| 6 | e | 0 | 55.759 |  | .160 | 824.586 | 1 | 1.143 |
| 7 | e | 0 | 53.562 |  | .088 | 823.801 | 1 | 1.088 |
| 8 | e | 0 | 53.436 |  | .014 | 823.781 | 1 | 1.022 |
| 9 | e | 0 | 53.655 |  | .001 | 823.781 | 1 | 1.001 |

##### Bootstrap (Default model)

##### Summary of Bootstrap Iterations (Default model)

##### (Default model)

| Iterations | Method 0 | Method 1 | Method 2 |
| --- | --- | --- | --- |
| 1 | 0 | 0 | 0 |
| 2 | 0 | 0 | 0 |
| 3 | 0 | 0 | 0 |
| 4 | 0 | 0 | 0 |
| 5 | 0 | 0 | 0 |
| 6 | 0 | 0 | 0 |
| 7 | 0 | 0 | 2 |
| 8 | 0 | 30 | 19 |
| 9 | 0 | 295 | 20 |
| 10 | 0 | 928 | 27 |
| 11 | 0 | 1106 | 3 |
| 12 | 0 | 922 | 3 |
| 13 | 0 | 567 | 3 |
| 14 | 0 | 312 | 0 |
| 15 | 0 | 184 | 0 |
| 16 | 0 | 87 | 0 |
| 17 | 0 | 60 | 0 |
| 18 | 0 | 45 | 0 |
| 19 | 0 | 387 | 0 |
| Total | 0 | 4923 | 77 |

0 bootstrap samples were unused because of a singular covariance matrix.

0 bootstrap samples were unused because a solution was not found.

5000 usable bootstrap samples were obtained.

##### Bootstrap Distributions (Default model)

##### ML discrepancy (implied vs sample) (Default model)

|  |  |  |
| --- | --- | --- |
|  |  | |-------------------- |
|  | 806.588 | |\* |
|  | 846.802 | |\* |
|  | 887.015 | |\*\*\* |
|  | 927.229 | |\*\*\*\*\*\* |
|  | 967.442 | |\*\*\*\*\*\*\*\*\*\*\*\* |
|  | 1007.656 | |\*\*\*\*\*\*\*\*\*\*\*\*\*\*\*\*\*\* |
|  | 1047.869 | |\*\*\*\*\*\*\*\*\*\*\*\*\*\*\*\*\*\*\*\* |
| N = 5000 | 1088.083 | |\*\*\*\*\*\*\*\*\*\*\*\*\*\*\*\*\*\* |
| Mean = 1055.239 | 1128.296 | |\*\*\*\*\*\*\*\*\*\*\*\*\* |
| S. e. = 1.153 | 1168.510 | |\*\*\*\*\*\*\*\* |
|  | 1208.723 | |\*\*\*\* |
|  | 1248.937 | |\*\* |
|  | 1289.150 | |\* |
|  | 1329.364 | |\* |
|  | 1369.577 | |\* |
|  |  | |-------------------- |

##### ML discrepancy (implied vs pop) (Default model)

|  |  |  |
| --- | --- | --- |
|  |  | |-------------------- |
|  | 862.004 | |\* |
|  | 882.043 | |\*\*\*\*\*\*\*\* |
|  | 902.083 | |\*\*\*\*\*\*\*\*\*\*\*\*\*\*\*\*\*\*\*\* |
|  | 922.122 | |\*\*\*\*\*\*\*\*\*\*\*\*\*\*\*\*\*\*\* |
|  | 942.161 | |\*\*\*\*\*\*\*\*\*\*\*\* |
|  | 962.200 | |\*\*\*\*\*\* |
|  | 982.239 | |\*\*\*\* |
| N = 5000 | 1002.278 | |\*\* |
| Mean = 924.391 | 1022.317 | |\* |
| S. e. = .450 | 1042.356 | |\* |
|  | 1062.395 | |\* |
|  | 1082.434 | |\* |
|  | 1102.473 | | |
|  | 1122.513 | |\* |
|  | 1142.552 | |\* |
|  |  | |-------------------- |

##### K-L overoptimism (unstabilized) (Default model)

|  |  |  |
| --- | --- | --- |
|  |  | |-------------------- |
|  | -403.308 | |\* |
|  | -300.023 | |\* |
|  | -196.738 | |\*\* |
|  | -93.453 | |\*\*\*\*\*\*\* |
|  | 9.833 | |\*\*\*\*\*\*\*\*\*\*\*\*\* |
|  | 113.118 | |\*\*\*\*\*\*\*\*\*\*\*\*\*\*\*\*\*\*\*\* |
|  | 216.403 | |\*\*\*\*\*\*\*\*\*\*\*\*\*\*\*\*\*\*\*\* |
| N = 5000 | 319.688 | |\*\*\*\*\*\*\*\*\*\*\*\*\*\*\* |
| Mean = 200.267 | 422.973 | |\*\*\*\*\*\*\*\*\*\* |
| S. e. = 2.641 | 526.258 | |\*\*\*\*\* |
|  | 629.543 | |\*\* |
|  | 732.828 | |\* |
|  | 836.114 | |\* |
|  | 939.399 | | |
|  | 1042.684 | |\* |
|  |  | |-------------------- |

##### K-L overoptimism (stabilized) (Default model)

|  |  |  |
| --- | --- | --- |
|  |  | |-------------------- |
|  | -97.234 | |\* |
|  | -51.466 | |\* |
|  | -5.697 | |\*\* |
|  | 40.071 | |\*\*\*\* |
|  | 85.839 | |\*\*\*\*\*\*\*\*\*\* |
|  | 131.607 | |\*\*\*\*\*\*\*\*\*\*\*\*\*\*\*\*\* |
|  | 177.375 | |\*\*\*\*\*\*\*\*\*\*\*\*\*\*\*\*\*\*\*\* |
| N = 5000 | 223.143 | |\*\*\*\*\*\*\*\*\*\*\*\*\*\*\*\*\*\* |
| Mean = 192.467 | 268.912 | |\*\*\*\*\*\*\*\*\*\*\*\*\* |
| S. e. = 1.187 | 314.680 | |\*\*\*\*\*\*\* |
|  | 360.448 | |\*\*\* |
|  | 406.216 | |\*\* |
|  | 451.984 | |\* |
|  | 497.753 | |\* |
|  | 543.521 | |\* |
|  |  | |-------------------- |

##### Model Fit Summary

##### CMIN

| Model | NPAR | CMIN | DF | P | CMIN/DF |
| --- | --- | --- | --- | --- | --- |
| Default model | 50 | 823.781 | 203 | .000 | 4.058 |
| Saturated model | 253 | .000 | 0 |
| Independence model | 22 | 3056.310 | 231 | .000 | 13.231 |

##### RMR, GFI

| Model | RMR | GFI | AGFI | PGFI |
| --- | --- | --- | --- | --- |
| Default model | .099 | .751 | .690 | .602 |
| Saturated model | .000 | 1.000 |  |  |
| Independence model | .307 | .353 | .292 | .322 |

##### Baseline Comparisons

| Model | NFI Delta1 | RFI rho1 | IFI Delta2 | TLI rho2 | CFI |
| --- | --- | --- | --- | --- | --- |
| Default model | .730 | .693 | .782 | .750 | .780 |
| Saturated model | 1.000 |  | 1.000 |  | 1.000 |
| Independence model | .000 | .000 | .000 | .000 | .000 |

##### Parsimony-Adjusted Measures

| Model | PRATIO | PNFI | PCFI |
| --- | --- | --- | --- |
| Default model | .879 | .642 | .686 |
| Saturated model | .000 | .000 | .000 |
| Independence model | 1.000 | .000 | .000 |

##### NCP

| Model | NCP | LO 90 | HI 90 |
| --- | --- | --- | --- |
| Default model | 620.781 | 536.109 | 713.005 |
| Saturated model | .000 | .000 | .000 |
| Independence model | 2825.310 | 2650.523 | 3007.444 |

##### FMIN

| Model | FMIN | F0 | LO 90 | HI 90 |
| --- | --- | --- | --- | --- |
| Default model | 3.282 | 2.473 | 2.136 | 2.841 |
| Saturated model | .000 | .000 | .000 | .000 |
| Independence model | 12.177 | 11.256 | 10.560 | 11.982 |

##### RMSEA

| Model | RMSEA | LO 90 | HI 90 | PCLOSE |
| --- | --- | --- | --- | --- |
| Default model | .110 | .103 | .118 | .000 |
| Independence model | .221 | .214 | .228 | .000 |

##### AIC

| Model | AIC | BCC | BIC | CAIC |
| --- | --- | --- | --- | --- |
| Default model | 923.781 | 933.869 | 1100.253 | 1150.253 |
| Saturated model | 506.000 | 557.044 | 1398.946 | 1651.946 |
| Independence model | 3100.310 | 3104.749 | 3177.958 | 3199.958 |

##### ECVI

| Model | ECVI | LO 90 | HI 90 | MECVI |
| --- | --- | --- | --- | --- |
| Default model | 3.680 | 3.343 | 4.048 | 3.721 |
| Saturated model | 2.016 | 2.016 | 2.016 | 2.219 |
| Independence model | 12.352 | 11.655 | 13.077 | 12.370 |

##### HOELTER

| Model | HOELTER .05 | HOELTER .01 |
| --- | --- | --- |
| Default model | 73 | 78 |
| Independence model | 22 | 24 |

##### Execution time summary

|  |  |
| --- | --- |
| Minimization: | .015 |
| Miscellaneous: | .370 |
| Bootstrap: | 5.458 |
| Total: | 5.843 |
